# External Responsiveness of the SuperOp^TM^ Device to Assess Recovery After Exercise: A Pilot Study

**DOI:** 10.3389/fspor.2020.00067

**Published:** 2020-07-14

**Authors:** Luca Paolo Ardigò, Stefano Palermi, Johnny Padulo, Wissem Dhahbi, Luca Russo, Simone Linetti, Drazen Cular, Mario Tomljanovic

**Affiliations:** ^1^Department of Neurosciences, Biomedicine and Movement Sciences, School of Exercise and Sport Science, University of Verona, Verona, Italy; ^2^Department of Public Health, University of Naples Federico II, Naples, Italy; ^3^Department of Biomedical Sciences for Health, Università degli Studi di Milano, Milan, Italy; ^4^Sport Science Program, College of Arts and Sciences, Qatar University, Doha, Qatar; ^5^Faculty of Psychology, University eCampus, Novedrate, Italy; ^6^Laboratory for Applied Physiology, Sport Systems, Talents Development and Influence of Physical Activities on Health, Faculty of Kinesiology, University of Split, Split, Croatia; ^7^Croatian Institute for Kinesiology and Sport, Split, Croatia; ^8^Einstein Craft for Research, Development, Education, Trade and Services, Split, Croatia; ^9^Faculty of Kinesiology, University of Split, Split, Croatia

**Keywords:** fatigue, physiology, endurance training, training load, recovery

## Abstract

Post-exercise recovery is a complex process involving a return of performance and a physiological or perceptual feeling close to pre-exercise *status*. The hypothesis of this study is that the device investigated here is effective in evaluating the recovery state of professional cyclists in order to plan effective training. Ten professional male cyclists belonging to the same team were enrolled in this study. Participants performed a 7-day exercise program [D1, D4, and D7: low-intensity training; D2 and D5: passive recovery; D3: *maximum* oxygen consumption (VO_2Max_) test (for *maximum* mechanical power assessment only); and D6: constant load test]. During the week of monitoring, each morning before getting up, the device assessed each participant's so-called Organic Readiness {OR [arbitrary unit (a.u.)]}, based on blood pressure (BP), heart rate (HR), features of past exercise session, and following self-perceived condition. Based on its readings and algorithm, the device graphically displayed four different colors/values, indicating general exercise recommendations: green/3 = “you can train hard,” yellow/2 = “you can train averagely,” orange/1 = “you can train lightly,” or red/0 = “you should recover passively.” During the week of research, morning OR values and Bonferroni *post-hoc* comparisons showed significant differences between days and, namely, values (1) D2 (after low intensity training) was higher than D4 (after VO_2Max_ test; *P* = 0.033 and *d* = 1.296) and (2) D3 and D6 (after passive recovery) were higher than D4 (after VO_2Max_ test; *P* = 0.006 and *d* = 2.519) and D5 (after low intensity training; *P* = 0.033 and *d* = 1.341). The receiver operating characteristic analysis area under curve (AUC) recorded a result of 0.727 and could differentiate between D3 and D4 with a sensitivity and a specificity of 80%. Preliminarily, the device investigated is a sufficiently effective and sensitive/specific device to assess the recovery state of athletes in order to plan effective training.

## Introduction

Post-exercise recovery is a complex process involving a return to performance and a physiological or perceptual feeling to near pre-exercise *status* (Kellmann et al., 2018) and has attracted widespread interest over the last 20 years (Ostojic, 2016). This process is made difficult by its multi-factorial nature and the varying timelines of its related and different variables (Minett and Duffield, 2014). However, high inter-individual variability and the speed of the recovery timeline also differs, often influenced by a variety of external (*viz*., training/match loads, sleep and nutrition) and internal factors (i.e., aerobic and intermittent-sprint capacities (Johnston et al., 2015). Hence, this is not easy to interpret and understand. Thus, the ability to identify faster or slower multifactorial recovery portraits may help the prescription of recovery strategies (Wilke et al., 2019). It is well known and often recommended that recovery time, appropriate recovery strategies, and training load should be individualized (Nédélec et al., 2012). In this context, identifying faster- and slower-recovery athletes may allow coaches to focus on smaller groups based on similar recovery characteristics (Doeven et al., 2018).

To reach a high level of performance, competitive cyclists must work hard to find a balance between the most appropriate training load and the following—minimal but adequate—recovery period (Lamberts et al., 2009). Whereas the training load can be influenced by several variables that can be planned (i.e., intensity, volume, frequency, and duration), recovery is influenced by less controllable factors such as stress, sleeping patterns, nutrition, and psychological comfort (Jeukendrup et al., 2000). If the training load is too high and/or recovery is not sufficiently effective, hard training cannot be tolerated to a great extent, and symptoms of fatigue develop quickly, which are difficult to overcome (Jeukendrup et al., 2000). A prolonged imbalance in this relationship leads to functional overreaching and can, in the long term, develop into overtraining syndrome, that can worsen performance further (Meeusen et al., 2006).

Literature contains several studies on the recovery assessment of athletes by means of wearables, but the validity of wearables has not always been assessed satisfactorily (Sperlich and Holmberg, 2017). One of the wearables for recovery assessment, available on the market is SuperOp™ (WELLNESS and WIRELESS SRL, Reggio Emilia, Italy). SuperOp™ is a tool that determines the extent to which an athlete's current homeostasis deviates from the ideal situation, thereby assessing his or her physical condition. Indeed, wearable training-monitoring technology is used widely nowadays. In fact, the distance from ideal homeostasis gives an indication of an organism's supercompensation phase and recovery after a training session (Saris, 2001). By monitoring two bloodstream variables, blood pressure (BP) and heart rate (HR), SuperOp™ aims to assess current homeostasis to provide practical suggestions for training planning. It claims to track the homeostasis trend by also taking into account certain external variables. First, SuperOp™ takes a picture of an athlete's homeostasis and then measures bloodstream variable changes to assess the metabolic stress level {the so-called Organic Readiness [OR] [arbitrary unit (a.u.)]}. Organic Readiness would indicate how far an athlete is from his or her ideal homeostasis and would define his or her physical condition. Assessment would be quick and reliable and would clarify in which phase of the supercompensation–recovery curve the athlete is at that present moment. To the best of our knowledge, no previous study has assessed SuperOp™ in evaluating a training athlete's OR. Therefore, the aim of the present study is to assess the effectiveness and sensitivity of SuperOp™ in evaluating the recovery state of professional cyclists to assist athletes and coaches in planning effective training.

## Materials and Methods

### Participants

Ten professional male cyclists (height 175 ± 9 cm, mass 65.4 ± 4.7 kg, BMI 21.4 ± 1.4 kg/m^2^, age 36.6 ± 0.3 years, *maximum* HR 183 ± 12 bpm, main training type endurance, sport cycling experience 11.4 ± 9.5 years, and weekly training 6 ± 1 day) belonging to the same team were enrolled in this study. Inclusion *criteria* were: (1) cyclists had to show operators to be experienced enough in using SuperOp™, (2) they had to show operators to be fully recovered from previous injuries, and (3) cyclists had a *maximum* oxygen consumption (VO_2Max_) test, administered by an expert sport scientist over the previous 6 months. All subjects signed a consent form after verbal and written explanations regarding the study were accepted. All methodological procedures were approved by the University of Split Ethics Committee.

### Methodology

**Figure 1:**
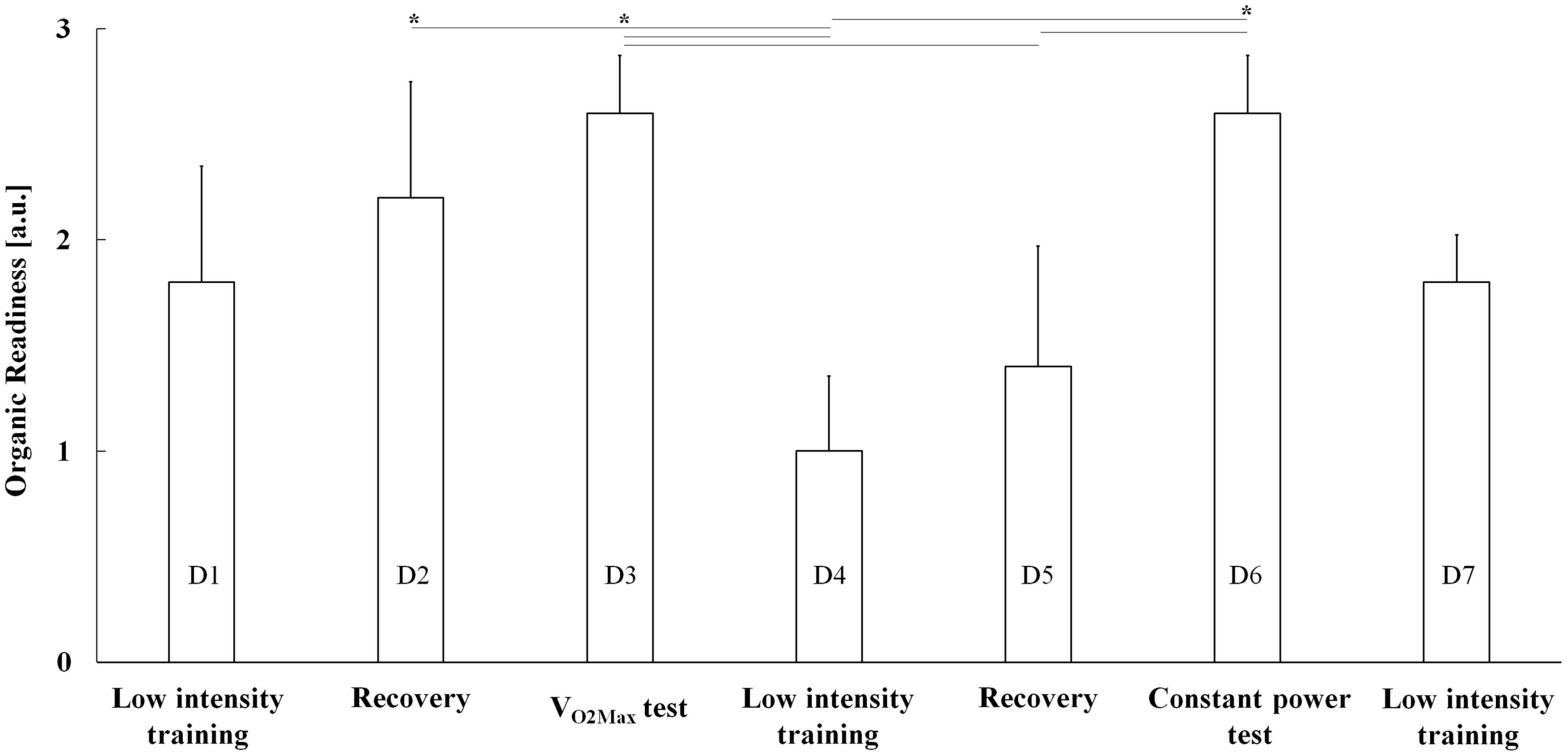
SuperOp™ Organic Readiness (OR) readings (mean + positive standard error) as a function of exercise days from D1 (low-intensity training) until D7 (low-intensity training). a.u., arbitrary unit, V_O2Max_ test, *maximum* oxygen consumption, *P < 0.05.

To assess SuperOp™'s (we obtained permission to use the device for our research from WELLNESS and WIRELESS SRL) effectiveness in evaluating the recovery state, we administered a 7-day exercise program, just after 3 days of very low-intensity training (Figure 1). All sessions were carried out at the same venue (Anima Sportiva allo Stato Puro, Palazzolo sull'Oglio, Italy; temperature 23.2 ± 0.6°C and relative humidity 55.3 ± 1.8%) and at the same time of the day (11 am) to avoid any circadian effects. The weekly schedule resulted as follows: on Day 1 (D1), low intensity training (@mechanical power corresponding to a value of 50% previously assessed VO_2Max_, to recover faster from previous workouts); Day 2 (D2), passive recovery; Day 3 (D3), VO_2Max_ test (for *maximum* mechanical power assessment only); Day 4 (D4), low intensity training (@mechanical power corresponding to a value of 50% of D3 VO_2Max_); Day 5 (D5), passive recovery; Day 6 (D6), constant load test (@mechanical power corresponding to a value of 80% of D3 VO_2Max_); and Day 7 (D7), low intensity training (@mechanical power corresponding to a value of 50% of D3 VO_2Max_).

SuperOp™ is intended as a training tool to support athletes in monitoring their training conditions. It consists of a device equipped with a blood pressure (BP) and heart rate (HR) wrist monitor (sensor Transtek LS810-B, Medaval Ltd, Dublin, Ireland) and has a Bluetooth-connected mobile phone application. A proprietary algorithm makes use of data featuring many past training sessions. Daily over our investigation, each morning (from 7 to 9 am) before getting up from their beds, athletes had to self-note down—from SuperOp™ readings—BP and HR. Furthermore, they had to self-put into SuperOp™ the previous day's exercise session data (time and duration) and the previous day's overall self-perceived condition (as colored emoticons: red when jaded or green when vivid). Finally, athletes had to self-note down—from consequent SuperOp™ readings—OR.

Organic Readiness (as four colors/values) is intended as being an outcome (i.e., recommendation), to establish a lower or higher body receptivity to a greater training load and the following positive benefit. Green/3 means “you can train hard,” yellow/2 “you can train averagely,” orange/1 “you can train lightly,” whereas red/0 means “you should recover passively.”

Each cyclist pedaled at the same relative mechanical power corresponding to its value at 50% VO_2Max_ on D1, D4, and D7 (“low-intensity training”). After warming up (8' at freely chosen gears/cadences), cyclists underwent a 40-min training session along a flat route, with their Polar kit-equipped bikes (different models, Pinarello, Treviso, Italy) setting their gears/cadences to ensure each individual target for mechanical power is maintained. Bikes were equipped with PowerTap P1 pedals (Charlie SRL, Arcugnano, Italy)—i.e., with power meter—allowing the continuous measurement of cadence and mechanical power. Low-intensity training was functional to athletes' recovery.

A *maximum* oxygen consumption test (for *maximum* mechanical power assessment only) was administered on D3. Each cyclist pedaled with his or her Polar kit-equipped bike (different models, Pinarello, Treviso, Italy) with PowerTap P1 pedals. Before the test, each cyclist warmed-up (8′ using gears/cadences of their choice). Cyclists underwent an up-to-exhaustion ramp protocol. Starting with free-wheeling, exercise intensity was increased by 25 W/min [conventional (upright, with approximately 75° trunk inclination with respect to horizontal, hands on handlebars, and elbows slightly bent) cycling posture and 70–90-rpm cadence, maintained by periodically self-checking the PowerTap P1 power meter handlebar-mounted display] (Padulo et al., 2015). The test ended when a cyclist spontaneously stopped pedaling or when he or she was unable to maintain a 70-rpm cadence). Measured variables were pre- and post-test (diastolic and systolic) BP, test average and maximum HR, pre-test and peak lactate concentration, and *maximum* mechanical power.

The “constant load test” session was administered on D6. After warming up (8' using gears/cadences of their choice), each cyclist pedaled for 40 min at a relative mechanical power corresponding to its value at 80% VO_2Max_ (i.e., equivalent to anaerobic threshold) along a flat route and with a Polar kit-equipped bike (different models; Pinarello, Treviso, Italy) with PowerTap P1 pedals, setting his or her own gears/cadences to maintain the mechanical power of his or her own individual target. Measured variables were pre- and post-test (diastolic and systolic) BP, test average and *maximum* HR, baseline and peak lactate concentration, and average mechanical power.

### Statistical Analysis

Statistical analysis was performed using the SPSS version 23.0 for Windows (SPSS Inc., Chicago, USA). Means and standard deviations were calculated after verifying the normality of distributions using the Shapiro–Wilk test. Therefore, parametric statistics was used. The variables analyzed were (morning) BP and OR. Regarding OR, it was calculated at 95% confidence intervals (95% CI), typical error of measure (TEM) (Atkinson and Nevill, 2000; Hopkins, 2000), and smallest worthwhile change (SWC) (Hopkins, 2000). If TEM is smaller than SWC, the ability of measure to detect small variable change is considered *good* (Liow and Hopkins, 2003). Subsequently, in order to determine any significant differences between different days, a repeated measure analysis of variance (ANOVA) was performed. When a significant *F*-value was found, the Bonferroni test was chosen as *post-hoc* test. Effect size was calculated as Cohen's *d*. A receiver operating characteristic (ROC) analysis was performed to calculate SuperOp™ sensitivity and specificity in discriminating only between D3 (after passive recovery) and D4 (after VO_2Max_ test), i.e., between the highest and lowest reading. Areas under curves (AUCs) were pooled, and heterogeneity was estimated using Q and I2 statistics. The Youden index was used to determine the *optimum* cut off point to differentiate D3 from D4 with acceptable sensitivity and specificity. The level set for significance was *P* ≤ 0.05.

## Results

**Figure 2:**
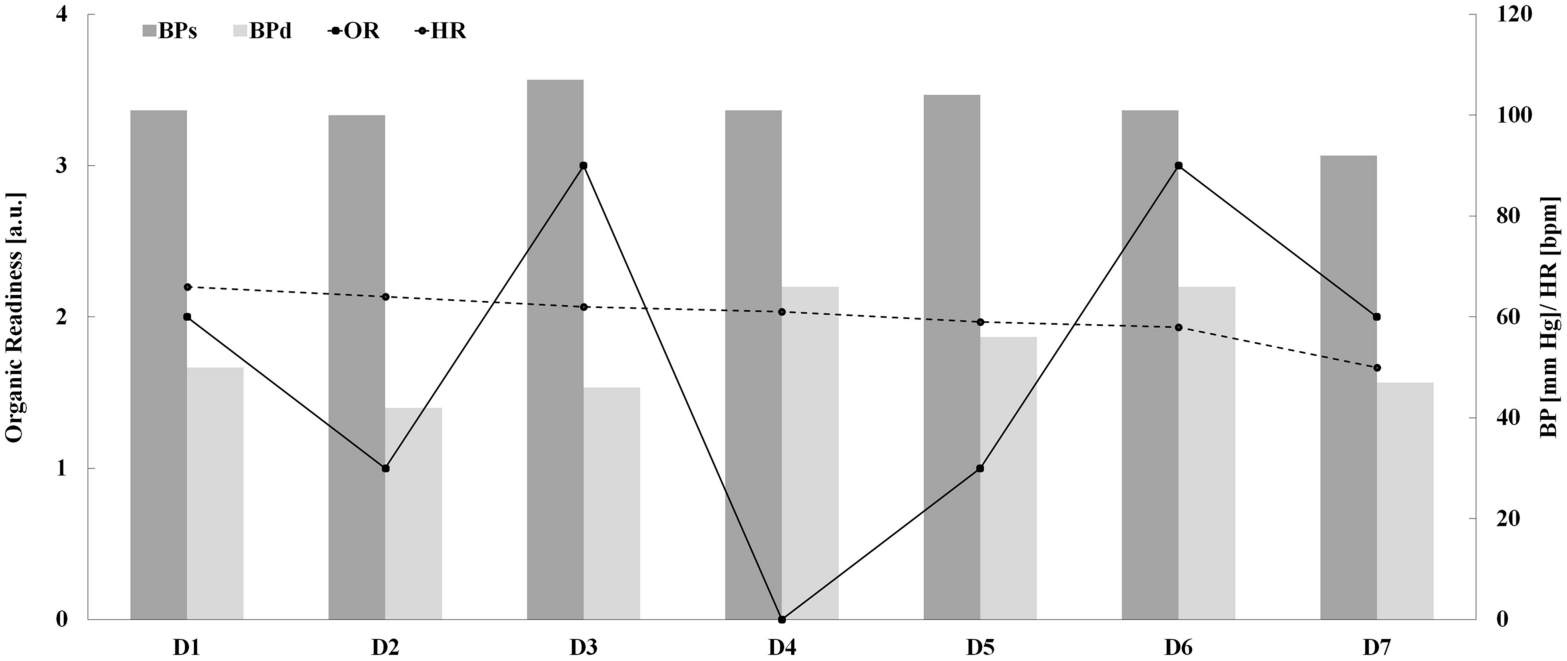
SuperOp™ Organic Readiness [OR (a.u.)], systolic [BPs (mm Hg)] and diastolic blood pressure [BPs (mm Hg)], and heart rate [HR (bpm)] readings as a function of exercise days from D1 until D7.

The main purpose of this study was to assess the effectiveness and sensitivity of SuperOp™ in evaluating professional cyclists' recovery state during a week of exercise. Morning OR, HR, and BP during the exercise week are shown in Table 1 and Figures 1, 2 (regarding a typical participant), whereas pre-, average-, and post-test (i.e., V_O2Max_ and constant load test) BP, HR, lactate, and mechanical power are shown in Table 2. Organic Readiness 95% CI, TEM, and SWC resulted in 0.4398–3.1602, 0.37, and 0.55 on D1; 0.8398–3.5602, 0.37, and 0.55 on D2; 1.9199–3.2801, 0.18, and 0.27 on D3; 0.1220–1.8780, 0.24, and 0.35 on D4; −0.0157–2.8157, 0.38, and 0.57 on D5; 1.9199–3.2801, 0.18, and 0.27 on D6; and 1.2447–3.3553, 0.15, and 0.22 on D7. With TEM being smaller than SWC in all days, the OR ability to detect small variable change resulted *good*. ANOVA showed significant differences over the week regarding OR [*F*_(1, 8)_ = 2.507 and *P* = 0.046], whereas no significant differences were found neither regarding HR [*F*_(1, 8)_ = 0.031 and *P* = 1.000] nor regarding systolic [*F*_(1, 8)_ = 0.108 and *P* = 0.995] and diastolic BP [*F*_(1, 8)_ = 0.305 and *P* =0.929; Table 1 and Figures 1, 2]. The *post-hoc* Bonferroni test showed significant differences regarding OR (1) between D2 and D4 (D2 > D4, *P* = 0.033 and *d* = 1.296) and (2) D3 and D6 compared with D4 (D3 and D6 > D4, *P* = 0.006 and *d* = 2.519) and D5 (D3 and D6 > D5, *P* = 0.033 and *d* = 1.341; Table 1 and Figures 1, 2). On D1, OR resulted in 1.80 ± 1.10, and accordingly, cyclists trained only at low intensity (Figures 1, 2; 167.0 ± 25.9 W). On D2, OR resulted in 2.20 ± 1.10, and accordingly, cyclists recovered passively. On D3, OR resulted in 2.60 ± 0.49, and accordingly, cyclists underwent VO_2Max_ test. On D4, OR resulted in 1.00 ± 0.71, and accordingly, cyclists trained again only at low intensity (168.0 ± 24.3 W). On D5, OR was 0.80 ± 0.40, and accordingly, cyclists recovered passively once more. On D6, OR was 2.60 ± 0.55 and accordingly, cyclists underwent a constant load test (Table 2) 298.0 ± 54.5 W). On D7, OR was 1.80 ± 0.55 and accordingly, cyclists trained again only at a low intensity (168.0 ± 24.3 W). AUC was 0.727 (95% confidence interval 0.432–0.923, *z*-statistic = 2.887, and *P* = 0.004) and could differentiate between D3 and D4 with a sensitivity and a specificity of 80% (Youden index = 0.455).

**Table 1:** Morning Organic Readiness (OR), heart rate (HR), and blood pressure (BP) over exercise week.

**Variables**	**D1**	**D2**	**D3**	**D4**	**D5**	**D6**	**D7**
Organic Readiness [arbitrary unit (a.u.)]	1.80 ± 1.10	2.20 ± 1.10	2.60 ± 0.55	1.00 ± 0.71^a^	1.40 ± 1.14	2.60 ± 0.55	1.80 ± 0.45
Heart rate (bpm)	47.2 ± 12.0	47.8 ± 10.7	47.4 ± 10.8	48.8 ± 8.8	48.4 ± 8.8	46.8 ± 9.0	47.0 ± 6.4
Systolic blood pressure (mm Hg)	114.4 ± 11.9	118.2 ± 11.5	116.0 ± 8.7	114.4 ± 9.9	116.8 ± 7.8	114.0 ± 7.6	114.8 ± 14.6
Diastolic blood pressure (mm Hg)	66.0 ± 6.9	64.2 ± 14.1	62.8 ± 9.8	66.6 ± 11.0	65.8 ± 9.0	70.8 ± 3.1	65.4 ± 10.7

*Data expressed as mean ± standard deviation. For Organic Readiness post-hoc Bonferroni test significant differences, refer to Figure 1. Other variables do not show any differences over exercise week*.

**Table 2:** Pre- and post-test blood pressures, average and *maximum* heart rates, pre- and post-test lactates, and mechanical powers.

**Variable**	***Maximum* oxygen**	**Constant**
	**consumption test**	**load test**
Pre-test systolic BP (mmHg)	125.8 ± 12.5	125.0 ± 10.6n.s.
Post-test systolic BP (mmHg)	142.2 ± 7.9	144.0 ± 8.0^**^
Pre-test diastolic BP (mmHg)	78.0 ± 11.5	73.6 ± 12.8^*^
Post-test diastolic BP (mmHg)	74.0 ± 8.5	79.6 ± 6.5^*^
Test average HR (bpm)	131.0 ± 19.1	142.0 ± 12.1n.s.
Test *maximum* HR (bpm)	185.0 ± 13.8	180.0 ± 11.9^*^
Pre-test lactate (mmol L^−1^)	1.3 ± 0.4	1.2 ± 0.3n.s.
Test peak lactate (mmol L^−1^)	12.7 ± 4.2	4.0 ± 0.2^**^
Test mechanical power (W)	360.0 ± 61.6^*^	298.0 ± 54.5^**^

*BP, blood pressure; HR, heart rate. Data expressed as mean ± standard deviation. n.s., not significant*,

* *P < 0.05*,

** P < 0.01 *maximum value.

## Discussion

The aim of this study was to preliminarily assess the efficacy of SuperOp™ effectiveness and sensitivity in evaluating the recovery state of professional cyclists' to assist coaches in planning effective training. The main indication of this study is that SuperOp™ is effective and sensitive in evaluating the physical recovery state of professional cyclists across a 7-day exercise program. This is documented by the significant differences among the OR values, recorded during the study period. Indeed, the morning after demanding exercises, OR values were lower (i.e., the recovery state was worse). The ROC curve analysis evidenced the sensibility of the SuperOp™ test in properly assessing the recovery state (to accurately provide future exercise recommendations) with 80% sensitivity and a specificity results.

It is well known that a physiological response to exercise is dependent on several variables (Burton et al., 2004). Nevertheless, we found that there was no significant difference between morning BP-values during the exercise week (Table 1 and Figure 2). BP changes during recovery could be due to modifications in regional and total peripheral resistance, cardiac output (stroke volume and/or heart rate), and plasma volume (Chen and Bonham, 2010). Although positive adaptative effects on several physiological variables, like BP, have been extensively proved in high-risk populations, a previous study (Biffi et al., 2018) also demonstrated BP adaptations in trained subjects (i.e., employees undergoing a 4-year corporate wellness supervised training program), followed over several years of training, such as the participants in the present study, which were experienced cyclists. Our results prove that BP alone does not affect the after-exercise recovery state; however, there is strong evidence in literature to indicate that post-exercise systolic BP is an additional risk marker for identifying asymptomatic individuals, with an increased risk of acute myocardial infarction (Kurl et al., 2001). Increased BP during exercise is a normal adaptation to effort within certain limits, but some studies reported that a blunted decline in systolic BP and elevated systolic BP after exercise are associated with an increased risk of coronary heart disease, stroke, and hypertension (Kurl et al., 2001). Although we did not monitor BP continuously during the VO_2Max_ test, recorded post-test values should be considered normal, although higher than pre-test values (Table 2). Moreover, a previous study (Sirico et al., 2019) showed excessively higher BP-values during an exercise stress test among a small percentage of the general population. Yet, the investigation of this variable did not form part of the focus of this study. However, in this study, each participant has been routinely tested for exercise stress adaptation and agonistic sport eligibility by his or her team's medical staff, with continuous blood assessments carried out during the exercise stress test, confirming the absence of any hypertensive subjects enrolled in this study.

We designed an experimental protocol to reproduce some common exercise intensities. We aimed at verifying how OR variables were sensitive to different bodies' recovery needs. In particular, the VO_2Max_ test was used as the most fatiguing exercise (Padulo et al., 2017). Further research could specifically investigate whether SuperOp™ could be used to evaluate the recovery state of professional athletes similar to the Total Quality Recovery Scale, (Osiecki et al., 2015).

Organic Readiness reading is also based on the previous day's training session data. Therefore, it is influenced by the athletes' condition each day and is, therefore, fundamental for athletes who train daily, such as weightlifters (Hartman et al., 2007). Such athletes have a crowded training/racing-recovery routine and, consequently, need to optimize the management of their time. Not only training but also an effective balance between training/racing and recovery is clearly crucial in maximizing an athlete's performance (Soligard et al., 2016). Nowadays, elite athlete training programs make use of a broad range of recovery-specific modalities such as massage or active recovery (Barnett, 2006). A device like SuperOp™ may support the different modalities. To the best of our knowledge, this is the first study to assess the ability of SuperOp™ to evaluate the recovery state of professional cyclists. Optimizing training for professional cyclists is an open challenge for sports professionals nowadays (Woods et al., 2018). Overuse and over-training can often be detrimental to competitive cyclists and, if untreated, can lead—at least—to delayed recovery (Faria et al., 2005). Past studies have shown that information on the recovery state of athletes can help plan a training schedule and preventing overtraining (Kenttä and Hassmén, 1998). It is already known that, because of individual differences, an optimal training load varies among athletes. Although recovery is theoretically important to improve performance (Richard and Koehle, 2019), evidence among cyclists with regard to this is lacking. SuperOp™ claims to be able to individually assess the recovery state of each individual athlete, and this could be a very practical utility for athletes and coaches. Although more research is needed on this topic, our study suggests that SuperOp™ is able to effectively and sensitively assess the recovery state of each individual athlete, serving as a means of preventing over-training (Halson and Jeukendrup, 2004).

The SuperOp™ device is easy to manage and is intuitive. The use of visual devices such as this may be of a great help for both athletes and coaches. By using similar devices, coaches can easily organize and manage their team's training. Devices like SuperOp™ may work well in combination with block periodization training (Rønnestad and Hansen, 2018).

This study has some limitations that should be taken into account. First, our results are encouraging but should be validated using a cohort larger than 10 subjects and an evaluation longer than 7 days. Investigating a larger sample may allow to better identify faster- and slower-recovery athletes to provide with stronger evidence coaches aiming at designing training programs differentiated for subjects with different recovery characteristics (Doeven et al., 2018). Moreover, SuperOp™'s use should be investigated in other sports and for longer periods, (e.g., during multi-stage cycling races such as Giro d'Italia, Tour de France, and Vuelta a España). An important question for future studies is to determine whether there are any differences regarding its use between amateur athletes and professional athletes. SuperOp™ may have the potential of being an essential tool in improving the sport training/racing-recovery cycle, but further work with regard to its functionality is needed. For instance, this study's ROC analysis was operated to calculate SuperOp™ sensitivity and specificity in discriminating only between the highest and the lowest reading. Intermediate readings should be investigated in the future. Yet, the study's main issue is the SuperOp™'s proprietary algorithm. Such an undisclosed calculation algorithm prevents the end user from being fully aware of the input data use in order to obtain a final OR value/recommendation. This is not a new issue among fitness devices (Chen and Bassett, 2005); therefore, due to this drawback of previous models, it is highly recommended that the SuperOp™'s manufacturer changes its research and development policy or at least significantly supports further validation of the device.

## Conclusions

SuperOp™ is a device aimed at evaluating an athlete's potential level of receptivity to a certain intensity of training, i.e., OR. Preliminarily, such a device shows good sensitivity in detecting the differences in the recovery state of professional cyclists over a 7-day exercise program. Therefore, it could support athletes and coaches in planning effective training. Nevertheless, we recommend that further larger studies be carried out in the future. When SuperOp™ indicated a red screen, passive recovery was recommended, whereas when a green screen was shown, hard exercising was recommended. The switch between red and green (and vice versa) was exactly as expected in our protocol (Table 1 and Figures 1, 2).

## Data Availability Statement

The raw data supporting the conclusions of this article will be made available by the authors, without undue reservation.

## Ethics Statement

The studies involving human participants were reviewed and approved by University of Split Ethics Committee. The patients/participants provided their written informed consent to participate in this study.

## Author Contributions

LA, SP, and JP share first authorship. All other authors contributed equally to this work. All authors contributed to the article and approved the submitted version.

## Conflict of Interest

The authors declare that the research was conducted in the absence of any commercial or financial relationships that could be construed as a potential conflict of interest.
